# α-synuclein pathogenesis in hiPSC models of Parkinson’s disease

**DOI:** 10.1042/NS20210021

**Published:** 2021-06-23

**Authors:** Jara M. Baena-Montes, Sahar Avazzadeh, Leo R. Quinlan

**Affiliations:** 1 Physiology School of Medicine, National University of Ireland Galway, Galway, Ireland; 2 CÚRAM SFI Centre for Research in Medical Devices, National University of Ireland Galway, Galway, Ireland

**Keywords:** α-synuclein, aggregation, iPSC, Parkinson's disease

## Abstract

α-synuclein is an increasingly prominent player in the pathology of a variety of neurodegenerative conditions. Parkinson’s disease (PD) is a neurodegenerative disorder that affects mainly the dopaminergic (DA) neurons in the substantia nigra of the brain. Typical of PD pathology is the finding of protein aggregations termed ‘Lewy bodies’ in the brain regions affected. α-synuclein is implicated in many disease states including dementia with Lewy bodies (DLB) and Alzheimer’s disease. However, PD is the most common synucleinopathy and continues to be a significant focus of PD research in terms of the α-synuclein Lewy body pathology. Mutations in several genes are associated with PD development including *SNCA*, which encodes α-synuclein. A variety of model systems have been employed to study α-synuclein physiology and pathophysiology in an attempt to relate more closely to PD pathology. These models include cellular and animal system exploring transgenic technologies, viral vector expression and knockdown approaches, and models to study the potential prion protein-like effects of α-synuclein. The current review focuses on human induced pluripotent stem cell (iPSC) models with a specific focus on mutations or multiplications of the *SNCA* gene. iPSCs are a rapidly evolving technology with huge promise in the study of normal physiology and disease modeling *in vitro*. The ability to maintain a patient’s genetic background and replicate similar cell phenotypes make iPSCs a powerful tool in the study of neurological diseases. This review focuses on the current knowledge about α-synuclein physiological function as well as its role in PD pathogenesis based on human iPSC models.

## Introduction

Neurodegenerative diseases are a group of progressive disorders characterized by neuronal cell death, excluding conditions primarily related to ischemia, infection or malignancy [1]. Neurodegenerative conditions are the most common age-related disorders in humans, becoming increasingly prevalent affecting millions of people worldwide. Despite significant scientific and clinical research effort, effective therapies are still lacking. Thus, it is vitally important to bridge the gaps in our understanding of the physiological and pathological processes underlying neurodegeneration to facilitate the development of targeted and effective treatment strategies. In the last 25 years, many cellular and molecular mechanisms have been identified that are associated with neuronal degeneration, most prominent among these are protein aggregate deposition [2], mitochondrial DNA mutations [3] and oxidative stress [4]. The formation of abnormal aggregates of physiological proteins has received great interest and is identified as a key hallmark for many neurodegenerative diseases, which are now grouped into what is termed as proteinopathies [5]. Neurodegenerative proteinopathies represent a group of diseases that are defined by inappropriate aggregation, deposition and/or accumulation of a normal protein that have a significant normal physiological function. Proteinopathies are classified based on the main protein found in these deposits, thus, tauopathies contain predominately τ protein and TDP-43 proteinopathies contain TDP-43 [6]. α-synuclein is a key member of this group of proteins involved in neurodegenerative disease.

α-synuclein has been shown to play a key role in the pathology of a variety of neurodegenerative conditions, grouped as synucleinopathies. α-synuclein is encoded by the *SNCA* gene which is found on chromosome 4 (4q21.3-22) and mutations in this gene show an autosomal dominant pattern of inheritance. Mutations in this gene have been shown to result in α-synuclein accumulation and aggregation which presents in many types of neurodegenerative conditions [7–9]. Well-known diseases such as Parkinson’s disease (PD), dementia with Lewy bodies (DLB) and multiple system atrophy (MSA) are captured in this group, as well as less common pathologies such as neuroaxonal dystrophies, pure autonomic failure (PAF) or REM sleep behavior disorder [10].

**Figure 1:**
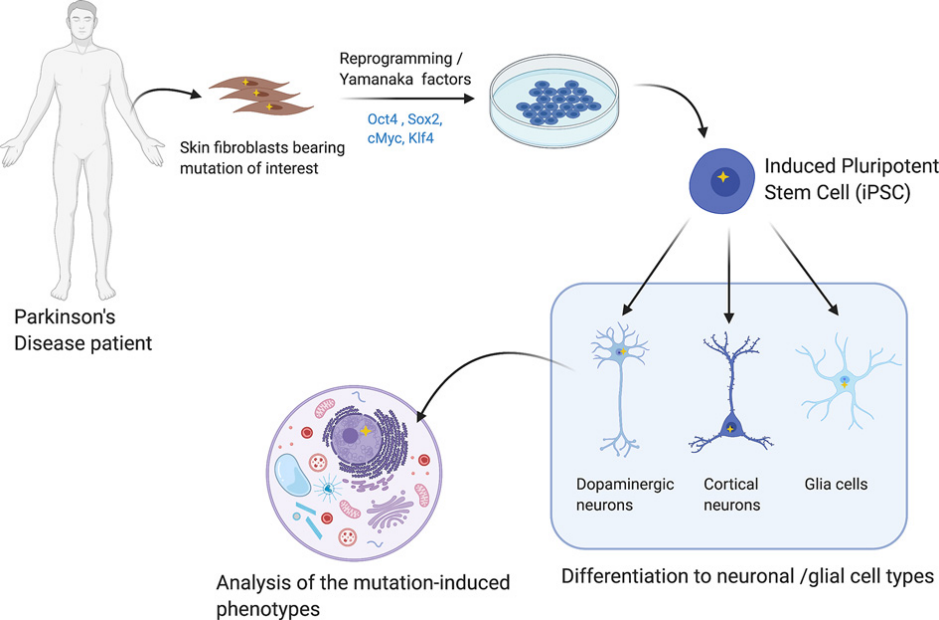
Parkinson’s patient iPSC-derived neurons procedure The fibroblasts are obtained usually from a skin biopsy from a Parkinson’s patient with a specific mutation (represented as a yellow star). The fibroblasts are reprogrammed *in vitro* into iPSCs and further differentiated to the cell of interest to study the mutation-induced phenotype.

Currently there is a broad spectrum of model systems available to aid in the study of synucleinopathies. Animal models provide valuable information about behavioral changes associated with neuronal alterations, but species differences create a barrier to obtaining human translatable disease-specific phenotypes. Cellular models have the advantage of allowing the pathology to develop rapidly, are cost effective and can be more easily genetically manipulated, gaining interest especially in molecular and cellular studies. In the last 14 years, the emergence of induced pluripotent stem cell (iPSC) technology has greatly advanced our understanding of patient-specific molecular mechanisms of disease, as well as the development of potentially new therapeutics and drug screening. This technology is based on the ability to reprogram disease-specific patient fibroblasts by forcing the expression of specific transcription factors (most commonly, Oct4, Sox2, cMyc and Klf4), resulting in a pluripotent state. Subsequently, these pluripotent cells are then differentiated to specific somatic mature cells of interest [11]. This type of approach is commonly known as ‘*disease in a dish*’ modeling [12] (Figure 1). This methodology has the advantage of maintaining the patient’s complete genetic background and allows the impact of certain key mutations on pathophysiology to be studied, allowing the characterization of key cellular mutation-based phenotypes in complex diseases such as PD [13].

Dopaminergic (DA) neurons are the main cell type used to study neurodegeneration in PD using several different protocols. Most protocols involve the forced expression of LMX1A, which encodes a transcription factor critical for ventral midbrain identity, taking a dual-SMAD inhibition approach. This process is based on the use of the compounds Noggin and SB431542 acting as inhibitors of the signal-transducer protein family SMAD (an acronym from the fusion of *Caenorhabditis elegans* SMA genes and the *Drosophila* MAD, Mothers against decapentaplegic), which are key regulators of cell growth [14–16]. More recently, differentiation can be directed by the forced overexpression of the factors ASCL1, NURR1 and LMX1A [17]. The reprograming of PD patient cells and differentiation into DA neurons has been reviewed extensively elsewhere [18,19].

Acknowledging the valuable information that iPSC models offer and the importance of α-synuclein in neurodegeneration, this review will focus on the knowledge gained from studying *SNCA* mutations in iPSC model systems, exploring α-synuclein aggregation and toxicity. In this context, some relevant questions will be discussed: are mutations in the *SNCA* gene the only instigator of α-synuclein aggregation? What is the pathogenic effect of *SNCA* mutations distinct from α-synuclein aggregation?

## α-synuclein: structure and normal physiological function

Based on the extant literature, α-synuclein is a 14-kDa protein, ubiquitously expressed in presynaptic terminals of the brain, predominantly in excitatory neurons, first reported in 1988 [20]. The native structure of the α-synuclein protein is still a source of debate, but is considered a natively unfolded protein under normal physiological conditions [21,22]. Thus its structure can vary according to changes in the local environment [23], where it may interact with lipids [24] or metals [25]. Changes in α-synuclein structure are thought to be related to its pathological misfolding and aggregation commonly seen in synucleinopathies [26]. For instance, the formation of α-synuclein oligomers induced by mutations such as E35K and E57K has been seen to affect permeability and integrity of the cell membrane promoting the death of the cell [27]. While many factors can contribute to aberrant α-synuclein production and aggregation, one of the main contributors are mutations of the *SNCA* gene which encodes α-synuclein and this gene was the first mutation reported in autosomal-dominant PD [28] with later association with DLB [8]. The precise physiological function of α-synuclein is still unknown but various roles associated with synaptic function have been identified. These functions include vesicle clustering, recycling and the maintenance of the synaptic vesicles reserve pool [29,30]. In addition, α-synuclein has been shown to promote SNARE complex formation which enhances neurotransmitter release [31]. In addition, it is also involved in intracellular trafficking regulation through interaction with multiple members of Rab GTPase family [32], as well as with microtubule nucleation and growth velocity [33]. Other studies based on data from PD brains, show that α-synuclein can also regulate dopamine levels by effecting DAT activity [34]. Increased levels of dopamine can lead to cell damage as a consequence of oxidative stress [35]. More recently, α-synuclein has been shown to inhibit phospholipase D (PLD) which is responsible for the conversion of phosphatidylcholine into phosphatidic acid, modulating neuronal processes such as growth, differentiation and the release of neurotransmitters and DA neurodegeneration [36,37]. α-synuclein has also been reported to play a role in neuroinflammation by initiating an immune response. Extracellular α-synuclein can trigger activation and proliferation of immune cells, cytokine secretion and phagocytosis [38,39].

## α-synuclein phenotype in *SNCA*-mutated iPSC-derived models

**Figure 2:**
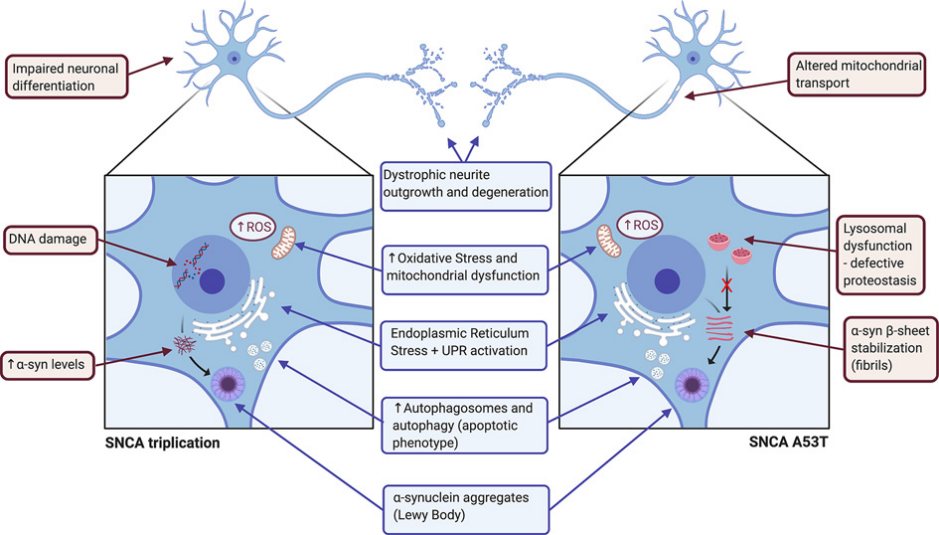
Summary of the cellular phenotypes reported in iPSC-derived neurons harboring *SNCA* triplication and A53T point mutation The common affected mechanisms for both mutations encompass impairment in neurite outgrowths, increased levels of oxidative stress and mitochondrial dysfunction, increased ER stress, imbalanced apoptosis and accumulation of α-synuclein aggregates, are shown in blue. The individual effects of the triplication of *SNCA* are impaired neuronal differentiation, DNA damage and increased levels of α-synuclein, whereas the ones of A53T mutation of *SNCA* are mitochondrial transport dysfunction, lysosomal activity impairment and accumulation of α-synuclein in fibrils, shown in red.

iPSCs offer several advantages over other model systems, with an unlimited supply of clinically relevant phenotypic cells of human origin while maintaining the patient’s original genomic features, including gene mutations or chromosome abnormalities. The main *SNCA* variants associated with genetic PD including the triplications/duplications [40] and missense point mutations like A53T [41], A30T [42] or E46K [9] have been modeled in iPSCs. Due to the high prevalence of triplications or A53T *SNCA* mutation in PD patients, the vast majority of iPSC models to date are focused on these two mutation types and their characteristic phenotypes are summarized in Figure 2.

### iPSC models of *SNCA* triplication

*SNCA* gene multiplication is associated with a younger age of PD onset and increased severity of symptoms. Triplications of *SNCA* result in generation of extra copies of the *SNCA* gene and overexpression of wildtype α-synuclein leading to the formation of toxic aggregates and widespread neuronal damage [43], suggesting a dose-dependent effect of α-synuclein in disease causation. *SNCA* triplication carriers present with a more severe phenotype and display a more rapid disease progression than duplication carriers and in many cases exhibit additional motor features [44]. Neuropathological examination of PD patient brains with *SNCA* triplication show severe degeneration of the substantia nigra, remarkable neuronal loss and vacuolation in the temporal cortex, as well as widespread Lewy body accumulation [45]. This pathology is mirrored in iPSC-derived DA neurons with *SNCA* triplication, which display increased α-synuclein mRNA levels, resulting in abnormal and elevated levels of protein expression [46]. In addition, iPSC-derived neurons harboring this mutation show higher levels of α-synuclein phosphorylation, something that is commonly found in PD brains [47], as well as abnormal increases in α-synuclein aggregates and Lewy bodies [9,48].

iPSC models are now also starting to provide additional information on the underlying molecular pathways with *SNCA* triplications. Endoplasmic reticulum (ER) stress and the activation of the unfolded protein response (UPR) is found to be activated in iPSC-derived neurons harboring *SNCA* triplication [49]. This demonstrates the crucial role the ER plays in the elimination of aberrant protein aggregates within the cell leading to ER stress and an associated UPR when ER capacity is exceeded.

Normal neuronal processes are affected by *SNCA* triplication and iPSC models have demonstrated that neuronal differentiation and maturation are altered by *SNCA* triplication. *SNCA* triplication iPSC-derived neurons are unable to generate a typical complex neuronal network, maintaining their proliferative capacity and display subtle changes in differentiation capacity. These alterations are further supported by the significant reductions observed in genes related to differentiation such as DLK, GABABR2 and NURR1, and a decrease in neurite outgrowth length [46,47]. These data point to a loss of regenerative capacity which may further compound the neuronal loss in PD patients.

Although α-synuclein is predominantly localized in presynaptic nerve terminals, a small fraction is also found in cell nuclei. iPSC neurons with *SNCA*-triplication show alterations in genome structure, resulting in DNA damage [50]. These iPSC-derived neurons express aberrant aging phenotypes as further evidenced by the decreased expression of heterochromatin markers and showing an abnormal nuclear envelope [48], as well as affecting genome integrity inducing DNA strand breaks and cell death [50].

Mitochondrial dysfunction is a common feature of neuronal loss and is the main organelle affected by α-synuclein pathology. In line with this, it is common to find mitochondrial impairment in iPSC-derived *SNCA* triplication neurons [51]. Mitochondrial impairment manifest as alterations in energy metabolism as a result of disruption in essential processes such as respiratory capacity and ATP production [52]. When *SNCA* triplication iPSC-derived neurons are exposed to low concentrations of the calcium ionophore ferutinin or laser-induced ROS, they have a higher susceptibility to permeability transition pores (PTPs) formation when compared with control neurons [53]. Several studies also demonstrate that *SNCA* mutations have increased basal sensitivity to toxin-induced oxidative stress which can be aggravated by metal ion interactions [54]. The exposure of *SNCA*-triplication iPSC-derived neurons to toxins such as 6OHDA results in increased cell death and caspase-3 activation [47] as well as an increase in autophagosomes [46]. These results are further supported by elevated levels of oxidative stress markers such as DNAJA1, HMOX2, UCHL1, HSPB1, involved in the protection of the cell against oxidative damage, and MAOA, which is a source of oxidative stress when overexpressed in these neurons [55].

### iPSC models of *SNCA*-A53T mutation

iPSC-derived neurons with the A53T mutation display higher tendency to produce α-synuclein oligomers and aggregates in comparison to control neurons. This maps well with what is observed in the human brain in patients carrying the same mutation [41,56]. The *SNCA*-A53T missense mutation was the first identified and is the most common mutation present in PD patients [28]. The A53T mutation is associated with an approximately 10-year earlier age of onset compared with other missense point mutations [44]. The A53T mutation stabilizes the α-synuclein protein in β-sheets, leading to a quicker rate of fibril formation as a toxic gain of function, contributing to the early onset of familial PD [26,57]. iPSC-derived neurons also show a dysregulation in protein production and transcription-related mRNAs due to the interaction of A53T mutated α-synuclein with essential transcription factors, ribonucleoproteins and ribosomal proteins, based on genome-wide analysis reports [58]. However, another study showed a decrease in tetramers/monomers ratio in *SNCA*-A53T iPSC-derived neurons compared with control suggesting that certain conformations such as tetramers may stabilize the protein and prevent the toxic effects observed with some oligomers [59].

As reported for *SNCA* triplication in iPSC-derived neurons, the UPR system is also disrupted in *SNCA*-A53T iPSC-derived neurons. This is associated with a reduction in the expression of the IREα factor, which is an essential component in this process [60]. The closely related pathway of lysosomal stress is also perturbed in A53T mutated iPSC-derived neurons, where α-synuclein binds and deactivates ykt6, resulting in protein aggregation that can be toxic to neurons [61].

Similar to the dystrophic neurite patterns observed in *SNCA* triplication neurons, this is also the case in *SNCA*-A53T iPSC-derived neurons [56]. Swollen varicosities and large spheroid inclusions, which are related to early neurite degeneration are present in *SNCA*-A53T iPSC-derived neurons. These alterations lead to the disruption in formation of neuronal networks with significantly reduces synaptic contacts [62]. Synaptic activity in *SNCA*-A53T iPSC-derived neurons is compromised with down-regulation of important pre- and postsynaptic cell adhesion proteins observed [62]. Moreover, the impairment of these processes leads to alteration in synaptic activity with larger mean amplitude on greater number of spontaneous Ca^2+^ transients [56].

In *SNCA*-A53T neurons, the anterograde mitochondrial transport process is disrupted which appears to be related to microtubule nitration and the inability to interact with mitochondrial transport complexes [63]. Similarly, *SNCA*-A53T iPSC-derived neurons show mitophagy delay related to up-regulation of Miro1, a key protein involved in mitochondrial transport [64]. Mitochondrial morphology is also altered to more circular and unbranched shape with a significant reduction in its membrane potential in mutated neurons [60]. Furthermore, antioxidant pathways are elevated, probably as a compensatory mechanism in response to the increase in mitochondrial stress. It has been speculated that this is due to increased levels of catalase or the peroxisome-proliferator activated receptor γ co-activator 1-α (PGC1-α) [60]. All these factors contribute to a pro-apoptotic phenotype that is present with the *SNCA*-A53T mutation. There is an increase in the expression of proteins related to autophagy, such as p62 or the autophagosome marker LC3 [60]. This process is especially aggravated in *SNCA*-A53T iPSC-derived neurons after exposure to agrochemicals [41].

## Additional factors influencing α-synuclein aggregation and pathology found in iPSC models

Although the presence of mutations in *SNCA* is a key factor that determines protein folding and aggregation into toxic species, other factors and variables have also been shown to play a role in this process. iPSC-derived neurons with mutations in other genes also show α-synuclein aggregation and display toxicity effects. iPSC-derived neurons bearing LRRK2 G2019S mutation present with increased levels of α-synuclein and have significant aggregations compared with controls [65]. Furthermore these neurons are sensitive to excessive degeneration when exposed to preformed α-synuclein fibrils (PFF). Interestingly, this effect was shown to be reversible, when the mutation was corrected in isogenic controls, aggregate formation was mitigated [66]. In addition, another factor influencing α-synuclein aggregation was found due to the differential expression of thioredoxin-interacting protein (TXNIP) in organoid cultures of iPSC-derived neurons with the LRRK2 G2019S mutation. TXNIP was previously identified as a risk factor for PD and its mutation and differential expression results in accelerated the accumulation of α-synuclein in LRRK2 G2019S neurons [67]. TXNIP mutations are also linked to deficits in autophagy mechanisms which contribute to increased levels of α-synuclein accumulation in neurons [68]. All these data are also in agreement with the evidence from human brain samples, which shows extensive α-synuclein pathology in PD patients with LRRK2 G2019S mutation [69].

The parkin gene (*PARK2*) encoding E3 ubiquitin ligase is another important factor in iPSC studies of α-synuclein. Recent studies show a significant elevation of α-synuclein levels and aggregation in iPSC-derived neurons from patients presenting with PARK2 mutations compared with control lines [70,71]. However, the absence of Lewy bodies in PD patient brains with parkin mutations make this detailed connection unclear, suggesting that parkin itself might interact and ubiquitinate the α-synuclein-interacting protein, synphilin-1 and promote the Lewy bodies inclusions [72]. There is also evidence of rare genetic risk factors for PD such as CHCHD2, showing an increase in insoluble α-synuclein accumulation in iPSC-derived DA neurons carrying CHCHD2 T61I mutation [73].

iPSC model systems have been invaluable in demonstrating these connections and highlight the utility and potential that iPSC technology can bring to the complex molecular mapping of α-synuclein neurodegeneration in PD.

## Limitations of iPSC models of disease models

Despite the many advantages that iPSC technology facilitates in disease modeling, there are still some limitations and challenges to overcome. Firstly, the most common challenge is the tumorigenicity that may be induced during the reprogramming process using retroviral and lentiviral reprogramming methods. The unknown or unmeasured effects of the reprogramming process are a potential confounding factor in assessing the truly representative nature of iPSCs as disease-specific models. However, it should be noted that more recent protocols use integration-free methods such as Sendai virus or DNA vectors and go some way to minimizing these problems [74,75]. Another hurdle that is well-known with stem cell studies is the intrinsic variability of iPSCs generated from different donors, or clones from the same donor, this variability is something that is difficult to reconcile in some instances as it may be a patient effect or a protocol effect. Reprogramming is designed to completely reset the epigenetic fingerprint of donor’s cells which in effect may lead to a biased differentiation potential into certain cell types [76], however some data appear to show that epigenetic memory is diminished over time in culture [77]. One of the principal limitations of iPSCs in relation to PD modeling is generating DA neurons with an aging phenotype. Studies have shown that the reprogramming process resets an aged cell to a more youthful state, with phenotypes having longer telomeres, reduced oxidative stress and competent mitochondrial organization [78,79]. Typically all cells use numerous quality control measures to protect normal physiological function, thus it is possible that phenotypic defects only manifest when protective pathways breakdown. Thus generating an aged phenotype is a complex task but some recent data suggest the possibility of inducing an aged phenotype by addition of progerin a truncated form of lamin A which is associated with premature aging [80], and telomerase inhibition [81]. Clearly there are some issues when using iPSC-derived neurons to model disease and particularly age-related disease states. Despite the challenges and potential pitfalls iPSC-derived neurons are a valuable resource in modeling α-synuclein pathology.

## Future directions with iPSC models of α-synuclein pathology

iPSC-derived neurons allow us to create a ‘*disease in a dish*’ but also facilitate the detailed study of the physiological pathways underlying disease states *in vitro*. α-synuclein aggregated species are found in the brains of most brain PD patients and iPSCs are a powerful tool to study the relationship between α-synuclein and neurodegeneration, exploring the physiological and pathophysiological roles of α-synuclein. The data from neuronal iPSC-derived models of specific genetic mutations associated with PD is growing and showing strong correlations with data from human brain samples [9]. Specifically in the case of *SNCA* mutations which are prevalent in the PD population, it is critically important that iPSCs as a model can strongly recapitulate the disease state. The data reviewed here suggest that iPSCs are indeed an excellent model to study physiology and pathophysiology of *SNCA* mutations.

Typically, *SNCA* mutations result in the stabilization and aggregation or fibrillation of α-synuclein in Lewy bodies together with other proteins. Once these aggregated species are present in the cell, they interact with other cellular structures such as microtubules, impairing axonal mitochondrial transport and ultimately leading to degeneration of the synaptic terminals and cell loss [9,26]. In addition, important mitochondrial functions are disrupted by α-synuclein oligomers’ interacting with ATP synthase such as the opening of PTPs, impairment in respiration and lipid peroxidation induction [53]. Moreover, α-synuclein aggregates’ interaction with proteins involved in mitophagy, and prevents the appropriate clearance of defective mitochondria from within the cell [64]. Interactions of α-synuclein oligomers with metal ions have also been suggested to induce the formation of free radicals in neurons, leading to the disruption of normal cell physiology, leading to cell death [54]. Most of the phenotypes displayed by iPSC-derived neurons are also found in the human brain, highlighting the suitability of iPSC modeling to not only in mimicking the cell physiological and pathological conditions but also their potential role as a platform to revealing novel data that might have previously relied on collecting brain biopsies from deceased patients.

Disease modeling with iPSCs has provided important supporting evidence that impairments in other cellular mechanisms can in some cases induce α-synuclein aggregation and accumulation. iPSC-derived neurons from PD patients bearing mutations, in LRRK2 or parkin highlight these interactions. For instance, ubiquitination of synphilin-1 in iPSC-derived neurons bearing parkin mutations is suggested to have an intermediate role inducing Lewy body formation [72]. Moreover, one of the key mechanisms that contribute to α-synuclein accumulation are defective autophagy and lysosomal proteolysis, which play a vital role in the clearance of defective aggregates. These processes are shown to be compromised in LRRK2 mutated iPSC-derived neurons [68,82]. In all these studies, iPSC-derived neurons display phenotypes that are closely aligned with that reported for human brain samples. Assessing the cause of α-synuclein aggregates commonly found in PD brains is complex and to date has proved unsuccessful.

While the definitive role of α-synuclein aggregation in PD pathology is still unclear, the literature shows a highly complex interaction between these aggregated species with many other proteins within the cell, creating a cascade of cellular pathway impairment that favors defective protein aggregation, ultimately leading to degeneration. In this broad and intricate molecular landscape, iPSC-derived models from PD patients can aid to identify the effect of the most common mutations in this pathology, being able to mimic the cellular processes of the PD brain with great precision. Moreover, this ‘*disease in a dish’* modeling system can facilitate both high throughput drug discovery and research into cellular therapy approaches. Future work with CRISPR-Cas9 technology in combination with iPSCs may revolutionize the approach to synucleinopathies with the aim of replacing the deleterious mutations or deleting the multiplications from the key disease genes [83] or indeed modulation of related mechanisms such as histones involved in post-translational modifications [84].

The extensive work carried out to date across multiple model systems, strongly suggests that the presence of α-synuclein aggregates, oligomers and fibrils have a central role in PD-related DA neurodegeneration. With an improving disease relevant platform base using iPSCs and the rapid growth in our understanding of the disease state, the future looks bright for therapies that can target synucleinopathies.

## Abbreviations

DA: dopaminergic
DAT: dopamine transporter
DLB: dementia with Lewy bodies
hiPSC: human induced pluripotent stem cell
PD: Parkinson’s disease
PTP: permeability transition pore
TXNIP: thioredoxin-interacting protein
UPR: unfolded protein response
6OHDA: 6-hydroxydopamine
